# White and Red Brazilian São Simão’s Kaolinite–TiO_2_ Nanocomposites as Catalysts for Toluene Photodegradation from Aqueous Solutions

**DOI:** 10.3390/ma12233943

**Published:** 2019-11-28

**Authors:** Lucas D. Mora, Larissa F. Bonfim, Lorrana V. Barbosa, Tiago H. da Silva, Eduardo J. Nassar, Katia J. Ciuffi, Beatriz González, Miguel A. Vicente, Raquel Trujillano, Vicente Rives, Maria Elena Pérez-Bernal, Sophia Korili, Antonio Gil, Emerson H. de Faria

**Affiliations:** 1 Grupo de Pesquisas em Materiais Lamelares Híbridos (GPMatLam), Universidade de Franca (Unifran), Av. Dr. Armando Salles Oliveira, 201 Parque Universitário, Franca-SP 14404-600, Brazil; moraengenharia@gmail.com (L.D.M.); labonfim96@gmail.com (L.F.B.); lorranavietro@yahoo.com.br (L.V.B.); eqhonorato@gmail.com (T.H.d.S.); eduardo.nassar@unifran.edu.br (E.J.N.); Katia.ciuffi@unifran.edu.br (K.J.C.); 2 GIR-QUESCAT, Dep. de Química Inorgánica, Universidad de Salamanca, E–37008 Salamanca, Spain; bei@usal.es (B.G.); mavicente@usal.es (M.A.V.); rakel@usal.es (R.T.); vrives@usal.es (V.R.); eperez@usal.es (M.E.P.-B.); 3 INAMAT, Departamento de Ciencias, Universidad Pública de Navarra, E–31006 Pamplona, Spain; sofia.korili@unavarra.es (S.K.); andoni@unavarra.es (A.G.)

**Keywords:** photodegradation, kaolinite, volatile organic compound, titanium dioxide, nanocomposites

## Abstract

The presence of volatile organic compounds in groundwater is a major concern when it is used as a drinking water source because many of these compounds can adversely affect human health. This work reports on the preparation and characterization of white and red Brazilian São Simão’s kaolinite-TiO_2_ nanocomposites and their use as catalysts in the photochemical degradation of toluene, a significant volatile organic compound. The nanocomposites were prepared by a sol-gel procedure, using titanium bis(triethanolaminate)diisopropoxide as a precursor. Thermal treatments of the nanocomposites led to different polymorphic titania phases, while the clay changed from kaolinite to metakaolinite. This structural evolution strongly affected the photocatalytic degradation behavior—all the solids efficiently degraded toluene and the solid calcined at 400 °C, formed by kaolinite and anatase, showed the best behavior (90% degradation). On extending the photochemical treatment up to 48 h, high mineralization levels were reached. The advantage of photodegradation using the nanocomposites was confirmed by comparing the results from isolated components (titanium dioxide and kaolinite) to observe that the nanocomposites displayed fundamental importance to the photodegradation pathways of toluene.

## 1. Introduction

With the population growth in recent years, there has been an increase in the generation and accumulation of industrial pollutants [1]. One of the current major problems is related to the most indispensable resource for all life on earth: water. According to United Nations (UN), one in three people do not have access to safe drinking water [2,3] and there is an urgent need for the development of effective removal techniques against the pollutants found in rivers, seas, lakes, etc. Advanced oxidative processes (AOPs) are among the most promising techniques for this purpose.

Volatile organic compounds (VOCs) are hazardous pollutants, being present in air and groundwater. Their release from numerous industrial sources, even in very low concentrations (<100 ppm), can cause health damage and their presence in groundwater used as a source of drinking water can cause serious health concerns, such as cancer and respiratory problems, among others [4]. The prediction of the environmental behavior of a given compound in groundwater depends on data that quantifies: (i) the compound’s tendency to volatilize (gaseous phase), (ii) its tendency to dissolve in water (aqueous phase), (iii) its tendency to float on or sink beneath the water surface, (iv) its tendency to dissolve in or to sorb other organic compounds (including natural organic matter), and (v) its affinity for ionically charged surfaces such as clay or soil particles [5]. Additionally, it is important to remark that VOCs dissolved in groundwater can react via hydrolysis, oxidation, hydroxylation, epoxidation, hydrogenolysis, halogenations, and other reactions which can lead to products with even increased toxicity [5].

According to Lawrence, toluene is present in groundwater, aquifers, domestic water, and public water, being ranked among the ten most widely detected VOCs in the United States [5]. For other countries, i.e., Brazil, similar data have not been reported. The solubility of toluene in groundwater is about 531 mg/L. 

Degradation of toluene has been reported by several authors. Thus, Fujihira et al. [6,7] studied the photo-oxidation of toluene in aqueous aerated suspensions containing various powdered semiconductors, reporting the formation of cresols, benzaldehyde and benzyl alcohol, depending on the pH of the solution and on the semiconductor used. The formation of benzaldehyde was also confirmed by Navío et al. [8], who used acetonitrile as a solvent and investigated the influence of the presence of water on product distribution. Recently, the use of a surfactant to enhance the photocatalytic reaction rate for toluene degradation has been reported [9]. The presence of surfactants or humic acids was found to be beneficial for degrading various substrates and a sequestration effect has been hypothesized for explaining this effect [10,11]. Marcì et al. [12] have reported the photocatalytic oxidation of toluene on irradiated TiO_2_, comparing the degradation performance in humidified air, in water, and in water containing a zwitterionic surfactant. A complete photo-oxidation of toluene was achieved after a few hours of irradiation in the presence of both types of catalysts—longer irradiation times produced the photodegradation of the surfactant.

As indicated, the use of AOPs is of great interest, as this group of techniques is able to successfully oxidize various organic compounds such as pesticides, herbicides, dyes, or drugs [13]. Among the AOPs, heterogeneous photocatalysis with TiO_2_ particles has been widely studied in recent decades for environmental remediation. This process has some interesting features: it occurs at room temperature, the required oxygen is taken from the atmosphere itself, the oxidation is complete to CO_2_, the photocatalyst has low cost and low selectivity, and can be reused [14]. The process begins with the incidence of UV radiation on the surface of titanium dioxide (anatase) and the generation of hydroxyl radicals that are able of oxidizing the organic pollutants, even completely mineralizing them.

To increase the specific surface area of the catalysts and their photocatalytic performance, silica, clay minerals or zeolite matrices have been used for the dispersion of TiO_2_ particles, increasing the number of active sites available for hydroxyl radical generation [3,5,15]. For instance, the formation of a titanium dioxide-hectorite composite with high titanium content (36 wt % Ti), showed an increase in specific surface area, adsorption capacity, and catalytic capacity for the photodegradation of VOCs. The amount of TiO_2_ on the hectorite surface was small, but the catalytic activity was also improved [16]. Chen et al. [17] prepared a series of silica-montmorillonite-titania photocatalysts and applied them to the degradation of single or mixed VOCs, finding excellent adsorption and photodegradation abilities for toluene, ethyl acetate (EA), and ethanethiol (EtSH). The adsorptive capacities of the catalysts increased in the order toluene < EA < EtSH. All kinetics using the catalysts followed the L-H model, regardless of being a single VOC or a mixture of them. The reaction rate constants for the photodegradation of the VOC mixture were all lower than those using only one VOC, in the order toluene < EA < EtSH. For the higher the adsorption capacity, the higher photocatalytic reaction rate, and 100% of all pollutants were removed separately.

On the other hand, Mishra et al. [18] quickly prepared TiO_2_-clay nanocomposites via microwaves, comparing their catalytic activity against photodegradation of methylene blue (MB) and chlorobenzene (CB). The photocatalytic efficiency was highly dependent on the clay structure (since a 2:1 clay (bentonite) showed higher activity than a 1:1 clay (kaolin)), in addition to their surface area and porosity. Throughout the study, the TiO_2_-kaolin composite was the least photoactive. Reactions with the TiO_2_/bentonite photocatalyst were faster than with other nanocomposites, with constant rates of 0.02886 min^−1^ and 0.04600 min^−1^ for MB and CB, respectively. 

Fan et al. [19] prepared nanocomposites of TiO_2_ on a reduced graphene oxide (RGO) matrix (with theoretical specific surface area near 2600 m^2^ g^−1^), employing them as efficient photocatalysts for hydrogen production from pure water and methanol aqueous solution. Zhang et al. [20] reported the selective oxidation of toluene and substituted toluene to the corresponding aldehydes over blank CdS, binary 5% RGO-CdS and ternary 5% RGO–10% TiO_2_-CdS composites under visible light irradiation. The 5% RGO-CdS composite displayed higher activity than blank CdS photocatalyst, while the introduction of TiO_2_ as second co-catalyst further improved the activity of CdS under identical reaction conditions [21].

Among the matrices used as supports, clay minerals stand out due to their low cost, high specific surface area, and ion exchange capacity. Thus, this work reports on the preparation and characterization of white and red kaolinite-TiO_2_ nanocomposites prepared by the sol-gel method using titanium (IV) bis(triethanolaminate)diisopropoxide as a precursor, and their use as catalysts in the photochemical degradation of toluene, an important VOC widely found in surface and groundwater.

## 2. Materials and Methods 

### 2.1. Clay Minerals Purification by Dispersion Decantation

The raw clay minerals (white and red kaolinites) were kindly supplied by the mining company Darcy R.O. Silva & Cia (São Simão, Brazil). Both clays were purified by the dispersion-decantation method to remove impurities [22,23]. The materials were named Kaol for white kaolinite and Kaol-R for red kaolinite. Their chemical compositions are given in Table 1.

### 2.2. Intercalation of Kaol and Kaol-R with Dimethylsulfoxide (DMSO)

As kaolinite is not easily swellable, it was previously expanded with dimethylsulfoxide (DMSO). The methodology used to intercalate the kaolinites with DMSO was based on procedures widely described in the literature [22,24], suspending purified kaolinite (Kaol or Kaol-R) in a mixture of DMSO and distilled water (1 g of clay/6.75 mL DMSO/0.75 mL water) for 10 days at 60 °C, in a reflux system and under constant magnetic stirring. The solids obtained were denoted as Kaol-DMSO and Kaol-R-DMSO.

### 2.3. Synthesis of Nanocomposite Photocatalysts

The catalytic materials were obtained by the sol-gel method, maintaining under magnetic stirring a mixture of the DMSO intercalated kaolinites (20 g), bis(triethanolaminate)diisopropoxide (5 mL) and isopropanol (150 mL), heated at 60 °C for 24 h. As no filtration was done during the preparation, all titanium may incorporate to the final solid, and with these amounts of reagents, the final amount of TiO_2_ should be 26 wt %. The materials were washed with ethanol and water and oven dried. The effect of calcination at three temperatures (400, 700 and 1000 °C, selected based on literature results [15]) was studied. After calcination the materials were named as Kaol-TiO_2_, Kaol-TiO_2_-400, Kaol-TiO_2_-700 and Kaol-TiO_2_-1000 for materials derived from white kaolinite; and Kaol-R-TiO_2_, Kaol-R-TiO_2_-400, Kaol-R-TiO_2_-700, Kaol-R-TiO_2_-1000 for materials derived from red kaolinite (for simplicity, “-DMSO” was omitted from the name of the final materials, although, as indicated above, they were prepared from the DMSO intercalated kaolinites).

### 2.4. Photodegradation of Toluene from Aqueous solution

All the photocatalysts were dispersed in aqueous solutions of toluene (0.05 g of photocatalyst in 5 cm^3^ of toluene solution with concentration of 20 mg L^−1^) and exposed to artificial ultraviolet radiation (λ = 365 nm, P = 30 W) for 24 or 48 h under constant stirring and placed in a thermostatic bath maintained at 25 ± 1 °C. The solutions were exposed to artificial UV light from a high pressure mercury vapor fluorescent lamp (OSRAM, Hns, UV-C emission at 365 nm, overlapping the absorption maximum of toluene), fixed horizontally at a distance of 25 cm from the center of the flask. The suspensions were then centrifuged, and the supernatants were collected for spectrophotometric analysis. Purified, un-functionalized, white and red kaolinite were also calcined at the same temperatures as the catalysts and used for comparative studies.

Determination of toluene in the original and supernatant liquid after reaction was carried out from the UV-vis spectra using a Hewlett-Packard UV-vis Spectrophotometer model 8453 (Agilent Technologies Brasil, São Paulo, Brazil), using quartz cells of 10-mm path length.

### 2.5. Photolysis of Toluene

A 20 mg L^−1^ solution of toluene was prepared in a 1000 mL volumetric flask and placed in a thermostat bath maintained at 25 ± 1 °C in the photoreactor chamber. The solution was continuously stirred with a gentle stream of air (100–120 bubbles per min) through a fine polythene tubing to ensure the uniform distribution of light throughout the solution. Samples of the photolyzed solutions were removed at suitable intervals for spectrofluorimetric assay. 

### 2.6. Measurement of Light Intensity

The amount of light emission under artificial and sun light was quantified by a INSTRUTEMP model LM801 luximeter (Instrutemp, São Paulo, Brazil), zero to 500000 lux range, and UV light was quantified by a UV light meter Instrutherm model MRU-201 (Instrutemp, São Paulo, Brazil), from 290 nm to 390 nm and quantification range from zero to 19990 µW/cm^2^, to verify the emission of UVA/UVB under photoreaction, with sun light exposure between 10:00 and 14:00. The values obtained are included in Table 2.

Experimental parameters under heterogeneous photocatalysis experiments, such as type of irradiation (artificial and sun light), no irradiation (dark experiments, adsorption), time of irradiation and toluene concentration, were evaluated to meet optimal experimental conditions. 

### 2.7. Characterization Techniques

Element chemical analysis of the parent kaolinites was performed at Activation Laboratories Ltd. (Ancaster, ON, Canada), using inductively coupled plasma-atomic emission spectroscopy (ICP-AES).

The powder X-ray diffraction (XRD) analyses of the solids were conducted on a Miniflex II Rigaku equipment (Rigaku Corporation, Tokyo, Japan), using Cu Kα radiation, λ = 1.54Å. The angle was varied between 3 and 75°, and all the samples were processed at a 2°/min rate following the powder method. 

Infrared (FTIR) absorption spectra were acquired on a Perkin Elmer FT-IR Frontier Spectrometer (Waltham, MA, USA) by using a diffuse reflectance accessory. In detail, 1 mg of each solid was mixed with 100 mg of KBr and finely pulverized until complete dilution. The pressed samples were analyzed by means of 32 scan acquisitions per spectrum and 1 cm^−1^ of nominal resolution.

The specific surface areas were determined by the BET method from the corresponding nitrogen adsorption isotherms (−196°C) measured in an ASAP 2020 physical adsorption analyzer from Micromeritics (Norcross, GA, USA). The samples (ca. 0.2 g) had been previously degassed for 1 h at room temperature at a pressure lower than 50 μm Hg.

Scanning electron microscopy (SEM) of the materials was performed on a Vega 3 SBH modelo EasyProbe from TESCAN digital scanning microscope (TESCAN, Brno, Czech Republic). The samples were previously coated with a thin gold layer by evaporation using a Bio-Rad ES100 SEN coating system (Bio-Rad Laboratórios do Brasil, São Paulo, Brazil).

UV-visible absorption of photocatalysts was determined by DRIFT-UV-Vis spectroscopy, using a portable Ocean Optics PX-2 apparatus (Ocean Optics, Amersham, UK), equipped with a QE 65000 detector (Ocean Optics, Amersham, UK), with diffuse reflectance accessory, both UV-Vis fiber with diameter 400 µm coupled to a solid state sample holder, ensuring the same measurement conditions for each sample (Ocean Optics, Amersham, UK). The spectra were collected with 2 nm of resolution, in the wavelength range between 200–1000 nm, and 32 scans were averaged. 

The cationic exchange capacity (CEC) of the clay was calculated by adsorption of methylene blue (MB), which also allowed to determinate the specific surface area (SSA) accessible to this molecule. An amount of 50 mg of the oven-dried sample was suspended in 10 cm^3^ of distilled water, and 0.5 cm^3^ aliquots of a 0.1 mol/L MB solution was added to the suspension with a volumetric burette (2.8 ≤ pH ≤ 3.8). After each addition, the suspension was homogenized by magnetic stirring for 1 min. Then, a small drop was removed from the solution and placed onto Fisher brand filter paper. The fact that non-adsorbed MB formed a permanent blue halo around the suspension aggregate spot on the filter paper meant that MB had replaced cations in the double layer and coated the entire surface. The cation exchange capacity was determined from the amount of MB required to reach the end point, according to the following Equation (1):

(1) $$CEC=\frac{\left[MB\right]\times V}{W}$$

where *CEC* is the cation exchange capacity (meq/100 g), [*MB*] is the concentration of the methylene blue solution (meq/L), *V* is the volume of the *MB* solution used during the assay (cm^3^), and *W* (g) is the mass of solid used in the experiment.

The specific surface area accessible to *MB* (*SSA*) was calculated according to Hang and Brindley [25] and Macek et al. [26]. This method assumes that *MB* molecules cover the particle surface area, and that the *MB* molecule approximates a rectangle with a surface area of 130 Å^2^/molecule. From the amount of adsorbed *MB*, expressed as *CEC* (Equation (1)), the *SSA* was calculated by means of Equation (2).

(2) $$SSA=F_{MB}\times CEC$$

where *SSA* is the accessible specific surface area (m^2^/g), *F_MB_* is a constant based on the approximated *MB* area, with a value 7.8043 (m^2^/meq), and *CEC* is the cation exchange capacity (meq/100 g).

## 3. Results and Discussion

### 3.1. Characterization of the Photocatalysts

**Figure 1:**
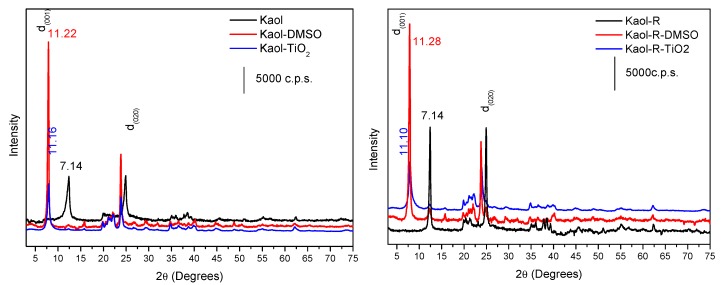
X ray powder diffractograms of the solids derived from Kaol (**left**) and from Kaol-R (**right**) kaolinites, at different preparation stages.

The chemical compositions of the parent kaolinites (Table 1) are close to the typical composition of this clay mineral. Normalized to 2 Al atoms, the following structural formulas were obtained: Si_2.5_Al_2_Fe_0.05_Mg_0.01_Mn_0.0003_K_0.02_Ti_0.05_O_8.20_ for white kaolinite and Si_2.4_Al_2_Fe_0.10_Mg_0.01_Mn_0.003_K_0.02_Ti_0.05_O_8.08_ for red kaolinite, respectively. In both cases, the formulas were close to the theoretical formula of kaolinite, Si_2_Al_2_O_7_, (also expressed as Si_2_Al_2_O_5_(OH)_4_ considering the hydroxyl groups). However, other elements were found in non-negligible amounts. First of all, Si can be mentioned, as the ratio Si/Al was 2.4–2.5, higher than the value of 2.0 in the pure clay. The presence of titanium (in very similar amounts in both clays), and of iron, twice as much in the red kaolinite than in the white kaolinite, probably influencing the color of the red solid, was also remarkable. Although much lower, the presence of manganese was also remarkable, mainly in the red solid. Some of these cations (Fe^3+^ and Mn^2+^) may be located in the octahedral positions of kaolinite, but in other cases the presence of mineral admixtures may be expected (as silica for the excess of Si, ilmenite or rutile for Ti, or other clay minerals for the alkaline or alkaline-earth elements), but they were not detected as crystalline phases, and only the kaolinite phase was found in the X-ray diffractograms of the solids (Figure 1). 

The basal spacing of the parent kaolinites was 7.14 Å for both solids. According to the X-ray diffractograms (Figure 1), the intercalation of Kaol and Kaol-R with DMSO successfully occurred, as reflections close to 7.89° were observed, corresponding to a basal spacing of 11.22 Å for Kaol and 11.28 Å for Kaol-R; the increase in the basal spacing confirmed the intercalation with DMSO [27,28,29]. After titanium (IV)triethanolaminate functionalization, a decrease in the basal spacing of the samples was observed, to 11.22Å and 11.10Å for Kaol and Kaol-R, respectively, evidencing that DMSO was replaced by the titanium alkoxide species.

**Figure 2:**
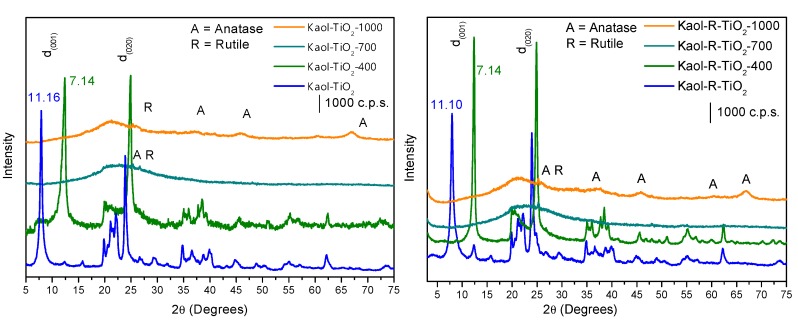
X ray powder diffractograms of the TiO_2_-containing solids derived from Kaol (**left**) and from Kaol-R (**right**) kaolinites, calcined at different temperatures.

The Kaol-TiO_2_ and Kaol-R-TiO_2_ solids had basal spacings of 11.16 Å and 11.10 Å, respectively (Figure 1), increased with respect to the original clays, which can be attributed to the incorporation of titanium oxyhydroxide species into the interlayer region. The presence in this region of DMSO (from the DMSO-kaolinite solids), triethanolamine (from the Ti-precursor) and isopropanol (from the reaction medium) should not be ruled out, but Ti-species derived from the hydrolysis of titanium (IV) triethanolaminate should be predominant. After heating treatments, the basal reflection disappeared, while TiO_2_ crystallized (Figure 2). Other kaolinite reflections were maintained at 400 °C, but for solids calcined at 700 and 1000 °C kaolinite was dehydroxylated, and its structure collapsed. Reflections from TiO_2_ anatase and rutile phases were close to those from kaolinite, making the identification difficult in the solid calcined at 400 °C, but for the solids calcined at 700 and 1000 °C the peaks from both titania polymorphs became evident [15,30].

Reinosa et al. [31] reported that the thermal treatment of kaolinite below 200 °C resulted in dehydrated kaolinite, near 500–580 °C the dehydroxylation of kaolinite led to metakaolinite phases (mixture of SiO_2_, Al_2_O_3_ and other oxides present as isomorphic substituents), while at temperatures higher than 980 °C mullite and amorphous silica were obtained (spinel phase). 

In our samples, it is important to remark that the calcination at high temperature may lead kaolinite to transform into metakaolinite (and if temperature is enough high, even to mullite), but at the same time, the predominant titania polymorph may change with temperature. Although they were present in low amounts, the evolution of the impurities present in kaolinite, isomorphically incorporated on its layers, was also interesting. Thus, the final catalytic behavior may be influenced by various factors, and this justifies the study of the evolution with temperature.

**Figure 3:**
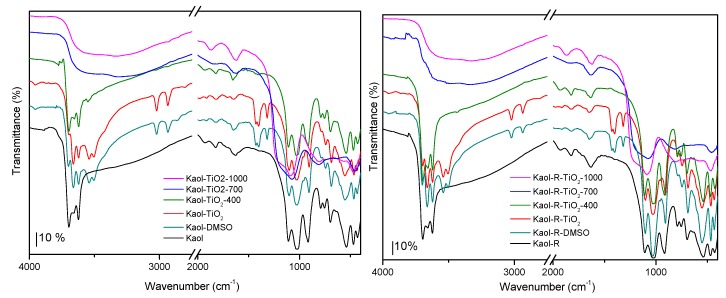
FTIR spectra of the solids derived from Kaol (**left**) and from Kaol-R (**right**) kaolinites, at different preparation stages (the high- and low-wavenumber regions are detailed in Figure S1).

The alterations in the FTIR bands due to inner surface hydroxyl evidenced the modifications in kaolinites. The interaction with DMSO became evident from the variations in the inner surface hydroxyl bands at 3696 and 3660 cm^−1^ (Kaol) and 3696 and 3658 cm^−1^ (Kaol-R) and in the inner hydroxyl bands at 3622 cm^−1^ for both solids (Figure 3 and Figure S1, Tables S1 and S2). Bands attributed to S=O–HO group, formed by the interaction of –S=O group from DMSO and clay hydroxyls, were observed at 3540 and 3504 cm^−1^, while bands from Al–OH shifted slightly, also proving the interaction [28,32]. After functionalization with the Ti-precursors, very low amounts of DMSO should remain in the solids, according to the very low intensity of the bands from this compound.

Upon heating at 400 °C, bands from the organic groups strongly decreased in intensity, although not completely disappearing, while bands from kaolinite were not altered. Calcination at 700 and 1000 °C led to the transformation from kaolinite into metakaolinite, also observing the vibration from amorphous silica in the 1200–1050 cm^−1^ region [15,27,30,33]. No bands involving Ti atoms were detected. Complete assignments of the bands are given in Tables S1 and S2.

**Figure 4:**
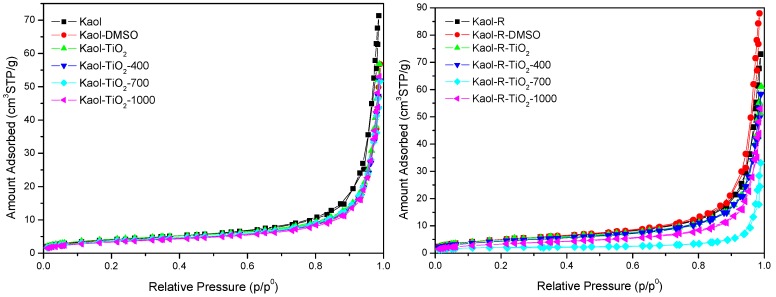
N_2_ adsorption-desorption isotherms of the solids derived from Kaol (**left**) and from Kaol-R (**right**) kaolinites, at different preparation stages.

The adsorption-desorption nitrogen isotherms for all solids were similar to each other (Figure 4), and belong to type III in the IUPAC classification [15,34]. This type of isotherm suggested a complex pore structure with undefined pore size and volume distributions due to the low adsorption values [35]. The BET specific surface area and pore volume for all the solids was low (Table 3). The values for the parent clays were typical for kaolinites. The intercalation of DMSO did not change the *S_BET_* and *V_p_* values, probably because all the molecules were removed during the outgassing step. Meanwhile, after treatment with the Ti-precursor, the *S_BET_* and *V_p_* values slightly decreased, probably because in this case the outgassing treatment was not able of completely remove the alkoxide moieties. Calcination should remove all the organic matter, and amorphous titanium phases should be formed, but at the same time the textural properties of the solids did not result improved, because of agglomeration of kaolinite platelets and its transformation into metakaolinite [15], and thus the final solids showed limited textural properties (8–16 m^2^/g and 0.051–0.090 cm^3^/g). 

The surface area accessible to *MB* is clearly higher than the *BET* values (Table 3). These differences are not unexpected, due to the different methodologies and intrinsic experimental conditions adopted in each case. The N_2_ method determines only the outer surface area, while the MB method measures the inner and outer surface area [36]. Values of *S_MB_* of ca. 60–80 m^2^/g were found, similar to values reported in the literature [15,30,37].

The band gap of the different solids was estimated by means of the Tauc plot approximation [38]. This method allows to determine the band edge from the UV-vis spectra of the solids by applying Equation (3):

(3) αhv=A(hv−Eg)12

where *α*, *h*, *ν*, *E_g_* and *A* denote the adsorption coefficient, the Planck constant, the radiation frequency, the band gap and a constant, respectively. From this equation, a plot of (*αhν*)^1/2^ *vs. hν*, the so-called Tauc plot, showed a linear region just above the absorption edge whose extrapolation to the photon energy axis (*hν*) provides the semiconductor band gap energy value. Immobilization of TiO_2_ into kaolinite only slightly modified the band gap (Table 3), probably because the high dispersion of TiO_2_ particles hindered the transference of energy into the environment. Various authors have determined that in TiO_2_ the rutile has a direct band gap of 3.06 eV and an indirect one of 3.10 eV and the anatase has only an indirect band gap of 3.23 eV [9,10,11,12].

**Figure 5:**
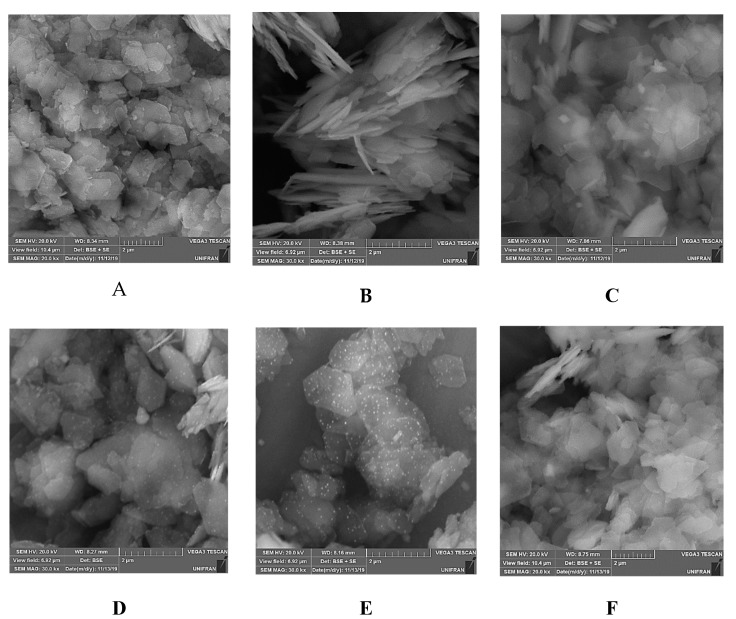
SEM analysis (BSE detector) of Kaol (**A**), Kaol-DMSO (**B**), Kaol-TiO_2_ (**C**), Kaol-TiO_2_-400 (**D**), Kaol-TiO_2_-700 (**E**), Kaol-TiO_2_-1000 (**F**). Magnification 30000x.

White and red kaolinite particles displayed face-to-face associations and their sizes varied between 1 and 2 µm (Figure 5). Pseudo-hexagonal kaolinite stacks and plates, also containing flakes mostly with broken edges, were observed along with the agglomerated particles [39]. The solids intercalated with DMSO showed more flake and irregular edges particles than parent clays. Kaol-TiO_2_ clearly showed a ‘‘spongy-like” aspect, which could be related to the presence of the oxide network incorporated into the clay. After the thermal treatment at 400 °C (Figure 5D), the nanostructured porous solid showed a similar aspect supporting that TiO_2_ nanoparticles were mainly incorporated into the kaolinite layers. A similar behavior took place in the Kaol-R-TiO_2_ photocatalysts (Figure S2A–E). The agglomeration of the smaller clay platelets was also observed after the incorporation of the Ti-precursor, as well as the presence of smaller irregular nanoparticles (white color), indicative of the entrance of the alkoxide into kaolinite layers (Figure S3A–E). Using the images obtained from backscattering electron imaging (*B_SE_*), the difference in contrast between kaolinite and TiO_2_ was enhanced, detecting the presence of decorated brighter titania particles around individual kaolinite particles, as confirmed by EDX analysis (Figures S4–S12). 

The small size of titania particles was very remarkable (Figure 5D,E). This strongly suggested that the titanium precursor used, bis(triethanolaminate) diisopropoxide, and its hydrolysis in the presence of kaolinite-DMSO, favored the formation of particles smaller than those obtained from other precursors and under different hydrolysis conditions [15].

### 3.2. Photocatalysis Experiments

#### 3.2.1. Preliminary Adsorption Tests

**Figure 6:**
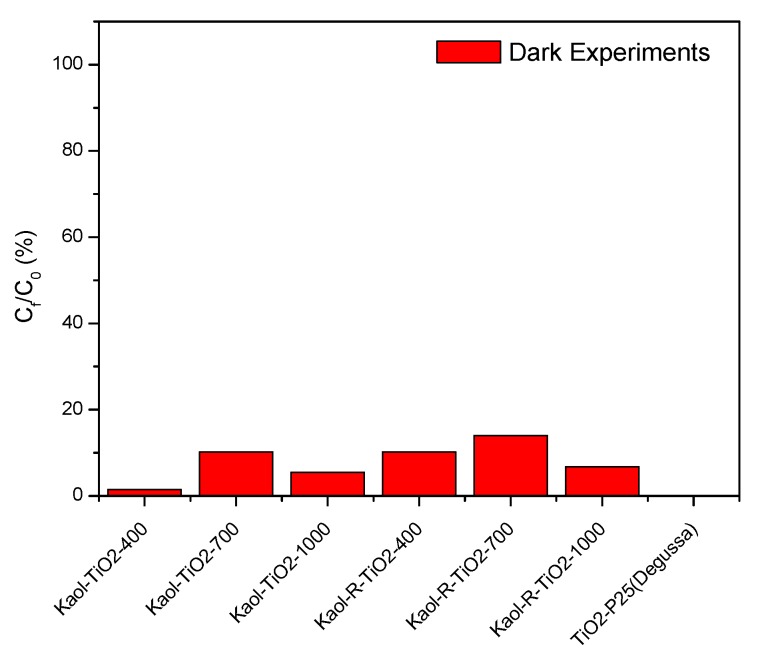
Dark experiments (adsorption) of toluene by synthesized white and red TiO_2_–kaolinite catalysts. For comparison, results for commercial TiO_2_-P25 (Degussa) are also included. Initial concentration of the toluene solutions: 20 mg L^−1^, mass of catalysts: 0.05 g, time: 48h.

Control experiments were carried out using the catalysts but in the absence of UV radiation, so-called adsorption tests. Low adsorption of toluene was observed (lower than 0.077 mg g^−1^, equivalent to ca. 1% of the existing toluene, even after 24 h, see Figure 6), probably related to the hydrophobic character of toluene, that strongly hindered its interaction with the very hydrophilic matrix and negatively charged layers of the original clays and the catalysts. However, the as-prepared solids, dried at only 100 °C, showed the typical profile of leaching of organic molecules to the solution (DMSO from the solids intercalated from this molecule, and isopropyl alcohol and ethanol after treatment with titanium bis(triethanolaminate)diisopropoxide). That is, the organic moieties remaining in the solids treated at low temperature leached into the solution, and as they absorbed in the region used for the determination of toluene, they caused an apparent increase in the amount of toluene. Thus, the apparent amount of toluene in the solution was almost twice as much as the initial one, although as indicated this is an experimental artifice.

#### 3.2.2. Photolysis and Blank Photocatalytic Tests

**Figure 7:**
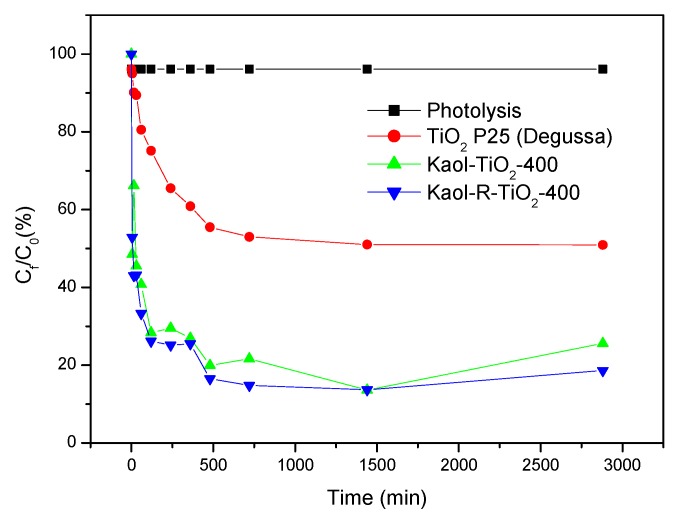
Photolysis of toluene and photodegradation using commercial TiO_2_-P25 (Degussa), kaolinite and red kaolinite treated at 400 °C as catalysts. Initial concentration of the toluene solution: 20 mg L^−1^, mass of catalysts: 0.05 g; artificial UV radiation: 365 nm, P = 30 W.

For evaluating the photolysis of toluene, a solution of this pollutant was submitted to UV radiation in the absence of catalysts. A very low degradation value, lower than 1%, was obtained, evidencing that toluene is not significantly degraded under UV radiation (see Figure 7).

Blank tests were also carried out using as catalysts the raw kaolinites and commercial TiO_2_–P25 (Degussa). Using the raw kaolinites calcined at 400 °C, the photodegradation profile reached a plateau after 4 h of reaction, removing 25% and 28% of toluene on Kaol-400 and Kaol-R-400, respectively. These results should be affected by the adsorption capacity of the clays, and also by the presence of small amounts of Ti and Fe, which could favor the reaction.

Commercial TiO_2_ showed high photocatalytic efficiency, with ca. 50% photodegradation of toluene after 2 h of reaction, remaining constant up to 48 h, proving the effect of the catalysts on the reaction. Marcì et al. [12] have reported the photocatalytic oxidation of toluene on irradiated TiO_2_, comparing the degradation performance in humidified air, in water and in water containing a zwitterionic surfactant. A complete photo-oxidation of toluene was achieved after a few hours of irradiation in the presence of both types of catalyst—longer irradiation times also produced the photodegradation of the surfactants.

#### 3.2.3. Photodegradation of Toluene Using the White and Red Kaolinite–Titanium Dioxide Catalysts

**Figure 8:**
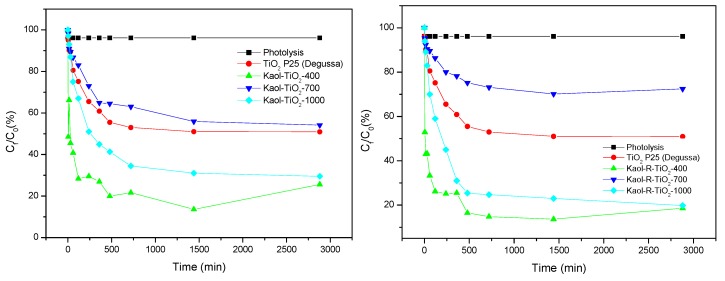
Kinetic profiles of toluene photodegradation experiments using the solids derived from Kaol (**left**) and from Kaol-R (**right**) kaolinites. For comparison, commercial TiO_2_-P25 (Degussa) and photolysis test are also included. Initial concentration of the toluene solutions: 20 mg L^−1^, mass of catalysts: 0.05 g; artificial UV radiation: 365 nm; P = 30 W.

The degradation of toluene by the white and red kaolinite–TiO_2_ catalysts, compared to the reference experiments described above, is also shown in Figure 7 and Figure 8. All the composites showed high degradation performance, even at short reaction times. At long reaction times, all the catalysts were able to degrade toluene, reaching values of 81% and 90% for Kaol-TiO_2_-400 and Kaol-R-TiO_2_-400, respectively.

The highest degradation profile was confirmed at 48 h. However, it is important to remark that the uncalcined solids did not show any degradation profile, the leaching of organic moieties from the solids induced an increase of absorbance, thus making the correct measurement of the values of toluene concentrations extremely difficult. Based on this point and on all characterization results that showed that the clay structure was maintained and TiO_2_ existed on the clay surfaces, the solids selected to evaluate the kinetic profile were those treated at 400 °C. It is important to remark that the solids calcined at 700 and 1000 °C also showed a high degradation profile, larger than 70 and 80%, respectively, also against toluene in aqueous solution (see Figure 7). These results were similar to those reported by other authors [40,41], where kaolinite–TiO_2_ composites were prepared by the hydrolytic sol–gel method and showed higher photo–degradation performance than TiO_2_-P25, as also reported for other photoprocesses [15,38]. It is necessary to highlight that the catalyst/toluene ratio used in this work was much lower than that used by other authors [15,40,41,42,43], so the degradation capacity of the prepared catalysts is much higher than that reported by other authors.

According to the results included in Figure 7 and Figure 8, the solids calcined at 400 °C provided the same degradation profile reaching the higher efficiency (90% within 480 minutes). The results for the solid derived from red kaolinite were slightly better than those derived from white kaolinite. From the data previously discussed, TiO_2_ nanoparticles deposited on the kaolinite surfaces may present a smaller particle size and lead to solids with a slightly larger surface area, such a dispersion of TiO_2_ nanoparticles resulting in the increase of the photodegradation performance. However, it is important to remark that these were not key factors affecting the catalytic properties, since, despite these discrete differences, the two solids had the same photodegradation efficiency.

A kinetic study of the catalytic photodegradation of toluene together with the estimation of the kinetic parameters is a necessary step in process design and optimization [44,45,46,47]. In the case of toluene photodegradation, few attempts have been performed. Hidaka et al. [48] reported the kinetic parameters of the Langmuir–Hinshelwood model obtained from the fitting of initial-rate values for one amount of catalyst; they also compared the degradation rate of dodecylbenzenesulphonate (DBS) with the degradation rate of various photoreactors employed. In addition to the Langmuir–Hinshelwood model, a first-order kinetic model (Equation (4)) was used to describe the degradation of several compounds [40,44,49,50]. In this work, experimental data were fitted to a first-order kinetic model.

The kinetic degradation of toluene was monitored and it was possible to calculate the reaction constant and the half-life time of the processes under study. The degradation time was related to toluene concentration in aqueous solution according to Equation (4):

*C* = *C*_0_·*e^−(k’·t)^* (4)

where *k’*= *k* [*SA*], *k* is the rate constant of the reaction, [*SA*] is the concentration of active sites on the catalyst surface, *t* is the irradiation time, and *Co* and *C* represent the concentration of toluene at the beginning of the reaction and with the time, respectively.

**Figure 9:**
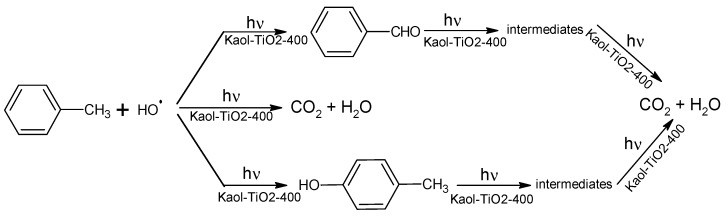
Mechanism proposed by Marcì et al. [12] for the formation of benzaldehyde and *p*-cresol and their intermediates and subsequent photo-mineralisation together with the pathway of direct photo-mineralisation of toluene catalysed by Kaol-TiO_2_-400 materials.

The formation of radical species responsible for toluene degradation was correlated to high rate constants of the reaction (*k’*) and low half-life times. However, for the constant *k’* to be high, which will affect the kinetics of degradation, the concentration of available active sites must also be high, since they are directly proportional to each other. Other forms of degradation can also occur by competitive mechanisms including the presence of catalytic active centres of other semiconductors related to the presence of Mn^2+^, Ti^4+^, Fe^2+^ and Fe^3+^ in the clay mineral as isomorphic substituents and did not follow the same mechanism when clays were used as matrices for degradation of toluene. The formation of benzaldehyde and *p*-cresol, and their subsequent photo-mineralisation together with the pathway of direct photo-mineralisation of toluene, can be roughly described by the following reaction mechanism, proposed by Marcì et al. [12] and adapted considering the solids prepared in this work (Figure 9):

The calculated kinetic parameters are included in Table 4. The reaction showed values of *k’* and *t_1/2_* very different for TiO_2_-P25 (Degussa). The values of *k’* and *t_1/2_* for the clay supports was slightly different, the Kaol-R derived solid showing higher *k’* and consequently smaller *t_1/2_* for toluene degradation, and therefore lower photodegradation rate and much higher *t_1/2_* for the Kaol derived sample. These results suggest that the occurrence of various photodegradation pathways not only depend on the interfaces, but also on the kind of catalyst and the support type used for immobilization of TiO_2_. The presence of TiO_2_ deposited on kaolinite surfaces increased the kinetic constant 50 times for Kaol and 90 times for Kaol-R solids, respectively, with respect to purified calcined clays Kaol and Kaol-R. This behaviour could be explained by the interactions between the active sites of the catalysts based on Kaol and Kaol-R and the adsorbed species. Benzoic acid and other intermediates can show high affinity to the surface of the catalyst, as previously described by Marcì et al. [12]. It has been reported that benzoic acid strongly interacted with the surface of TiO_2_, as evidenced by the FTIR spectroscopy results, and it was subsequently degraded without being released to the liquid phase [12]. 

**Figure 10:**
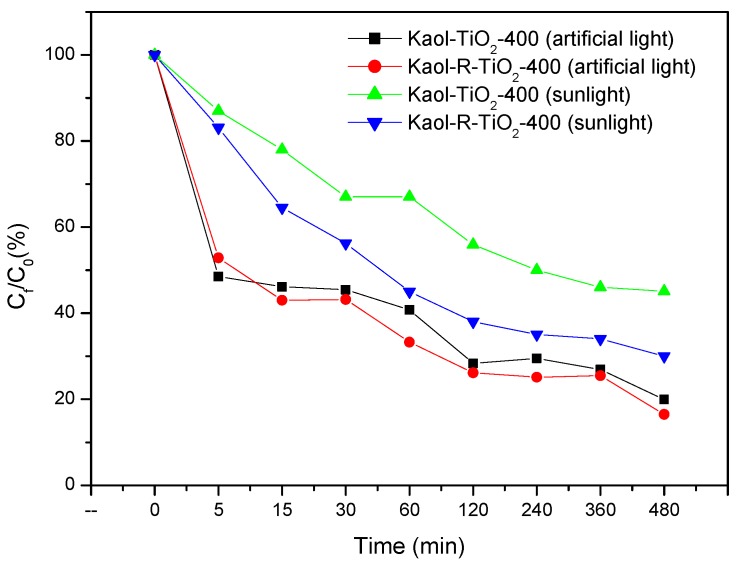
Kinetic profiles of the photocatalysis experiments of toluene photodegradation by synthesized white and red TiO_2_–kaolinite catalysts using sunlight for photoreactions compared with artificial light experiments. Initial concentration of the toluene solutions: 20 mg L^−1^, mass of catalysts: 0.05 g.

Our results showed that photodegradation of toluene could occur under sunlight in the presence of the catalysts based on white and red kaolinite suggesting a degradation mechanism different from that the typically suggested partial hydrolysis in water (Figure 10).

The experiments with solar irradiation showed the typical profile obtained under an artificial photoreactor chamber (Figure 10), and red kaolinite showed higher photodegradation efficiency than white kaolinite, suggesting that the presence of dissolved oxygen acting in these reactions could not be ruled out. The degradation in both cases was not complete (56% and 70%, respectively, for Kaol-TiO_2_-400 and Kaol-R-TiO_2_-400). This efficiency was very similar compared to that of artificial light when comparing the irradiation doses. The small decrease of efficiency, 15%, observed with sunlight-induced reactions was explained by the efficiency of light absorption on the system. Removal kinetics profiles improved in both cases under solar light irradiation for red kaolinite compared to white kaolinite, although the photon flow needed for significant toluene degradation was far from the typical applied doses under artificial light (photoreactor).

## 4. Conclusions

Heterogeneous catalysts using white and red kaolinites as supports were prepared for application to toluene photodegradation in an aqueous solution. TiO_2_ deposited on the layered type solids promoted the good dispersion of semiconductors around clay layers and this class of material elicited great interest. Toluene photodegradation was more efficient using the solid treated at 400 °C that maintained the layer integrity of kaolinite and the other heat-treated samples also photodegraded toluene under artificial light, providing an alternative path for VOC photodegradation. Control reactions performed without catalysts (photolysis) did not show any toluene degradation, and adsorption did not occur either due to the high hydrophobic nature of the substrate. The results obtained suggest a photo-induced mechanism based on the photoionization of excited toluene by electron transfer to dissolved oxygen, and the subsequent formation of superoxide and hydroxyl radicals. Photodegradation using sunlight confirmed the applicability of these solids as efficient catalysts. All this, together with the low price and high availability of kaolinite, give these catalysts high interest. 

## Figures and Tables

**Table 1:** Chemical composition of white and red São Simão kaolinites (wt %, water-free form).

Sample	SiO_2_	Al_2_O_3_	Fe_2_O_3_	MnO	MgO	CaO	Na_2_O	K_2_O	TiO_2_
Kaol	55.90	43.72	1.12	0.007	0.27	0.09	0.06	0.44	1.14
Kaol-R	52.37	43.26	2.24	0,064	0,24	0.02	0.01	0.39	1.15

**Table 2:** Quantification of UVA/UVB (µW/cm^2^), and intensity and of luminosity (lux).

Exposition Site	Sun Light		Photoreactor	
	UVA/UVB	Luminosity Intensity	UVA/UVB	Intensity of Luminosity
Photoreactor (artificial ultraviolet radiation (λ = 365 nm, P = 30 W). Lowest and highest values measured	-	-	7–20	1548–1650
Indirect exposure (under shadow)	530	13,470	-	-
Direct exposure	1628	501,000	-	-
Laboratory, ambient quantification	-	-	1	800–949

**Table 3:** Specific surface area accessible to nitrogen (*S_BET_*) and to methylene blue (*S_MB_*), pore volume and band gap of the different solids.

Solid	*S_BET_* (m^2^/g)		*S_MB_* (m^2^/g)		*V_P_* (cm^3^/g)		Band Gap (eV)	
	Kaol	Kaol-R	Kaol	Kaol-R	Kaol	Kaol-R	Kaol	Kaol-R
Kaol	15	18	78	62	0.110	0.113	4.51	4.49
Kaol-DMSO	13	18	78	62	0.088	0.136	4.71	4.52
Kaol-TiO_2_	15	17	47	62	0.088	0.094	4.67	4.71
Kaol-TiO_2_-400	13	16	78	62	0.080	0.090	4.92	4.72
Kaol-TiO_2_-700	13	14	78	78	0.079	0.051	4.76	4.74
Kaol-TiO_2_-1000	14	12	62	62	0.081	0.082	4.79	4.85

**Table 4:** First order kinetic parameters obtained from experimental results of photodegradation of toluene in 20 mg L^−1^ aqueous solution using Kaol-TiO_2_-400 and Kaol-R-TiO_2_-400 as photocatalysts.

Catalysts	*k* (min^−1^)	*R* ^2^	*t*_1/2_(min)	*Х* ^2^
Kaol-TiO_2_-400	0.017	0.81	40	0.190
Kaol-400	0.0003	0.43	2074	0.080
Kaol-R-TiO_2_-400	0.034	0.62	20	0.370
Kaol-R-400	0.0004	0.62	1848	0.104
TiO_2_ P25 (Degussa)	0.0010	0.69	728	0.158

## Supplementary Materials

The following are available online at https://www.mdpi.com/1996-1944/12/23/3943/s1, Figure S1. Detail plot of the high- and low-wavenumber region of the FTIR spectra of the different solids; Figure S2. SEM analyses (BSE detector) of Kaol-R, Kaol-R-DMSO, Kaol-R-TiO_2_, Kaol-R-TiO_2_-400, Kaol-R-TiO_2_-700, Kaol-R-TiO_2_-1000. Magnification 30000X; Figure S3. SEM analysis (BSE detector) of Kaol, Kaol-DMSO, Kaol-TiO_2_, Kaol-TiO_2_-400, Kaol-TiO_2_-700, Kaol-TiO_2_-1000) (Magnification 5000X) and Kaol-TiO_2_-700 (Magnification 30000X); Figure S4. EDX spectrum of Kaol-TiO_2_; Figure S5. EDX spectrum of Kaol-TiO_2_-400; Figure S6. EDX spectrum of Kaol-TiO_2_-700; Figure S7. EDX spectrum of Kaol-R-TiO_2_; Figure S8. EDX spectrum of Kaol-R-TiO_2_-400; Figure S9. EDX spectrum of Kaol-R-TiO_2_-700; Figure S10. EDX spectrum of Kaol-R-TiO_2_-1000; Figure S11. EDX mapping of Kaol derivatives; Figure S12. EDX mapping of Kaol-R derivatives; Table S1. FTIR assignments (cm^−1^) of the solids derived from white kaolinite; Table S2. FTIR assignments (cm^−1^) of the solids derived from red kaolinite.
