# Supplementary material for: White and Red Brazilian São Simão’s Kaolinite–TiO2 Nanocomposites as Catalysts for Toluene Photodegradation from Aqueous Solutions
Source: Materials (Basel). 2019 Nov 28;12(23):3943. doi: 10.3390/ma12233943 (PMC6926498; doi:10.3390/ma12233943)
Supplement: Supplementary file 1 [file materials-12-03943-s001.pdf]

materials

MDPI

Article

White and Red Brazilian São Simão's Kaolinite–TiO_2 Nanocomposites as Catalysts for Toluene Photodegradation from Aqueous Solutions

Lucas D. Mora [1], Larissa F. Bonfim [1], Lorrana V. Barbosa [1], Tiago H. da Silva [1], Eduardo J. Nassar [1], Katia J. Ciuffi [1], Beatriz González [2], Miguel A. Vicente [2], Raquel Trujillano [2], Vicente Rives [2], Maria Elena Pérez-Bernal [2], Sophia Korili [3], Antonio Gil [3] and Emerson H. de Faria [1,*]

1 Grupo de Pesquisas em Materiais Lamelares Híbridos (GPMatLam), Universidade de Franca (Unifran), Av. Dr. Armando Salles Oliveira, 201 Parque Universitário, Franca-SP, 14404-600 Brazil
2 GIR-QUESCAT, Dep. de Química Inorgánica, Universidad de Salamanca, E–37008 Salamanca, Spain
3 INAMAT, Departamento de Ciencias, Universidad Pública de Navarra, E–31006 Pamplona, Spain
* Correspondence: emerson.faria@unifran.edu.br; Tel.: +55-16-3711-8969

Received: 30 October 2019; Accepted: 26 November 2019; Published: 28 November 2019

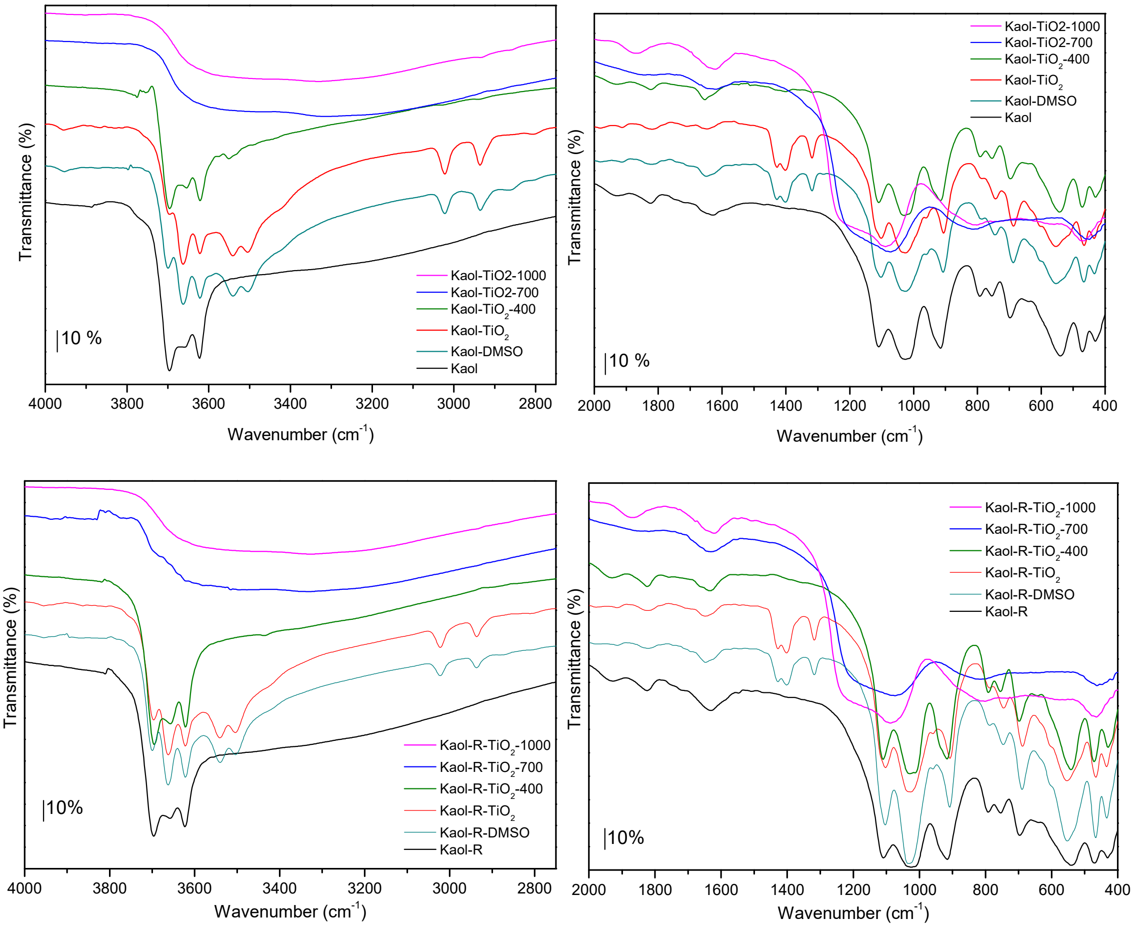

Figure S1. Detail plot of the high- and low-wavenumber region of the FTIR spectra of the different solids.

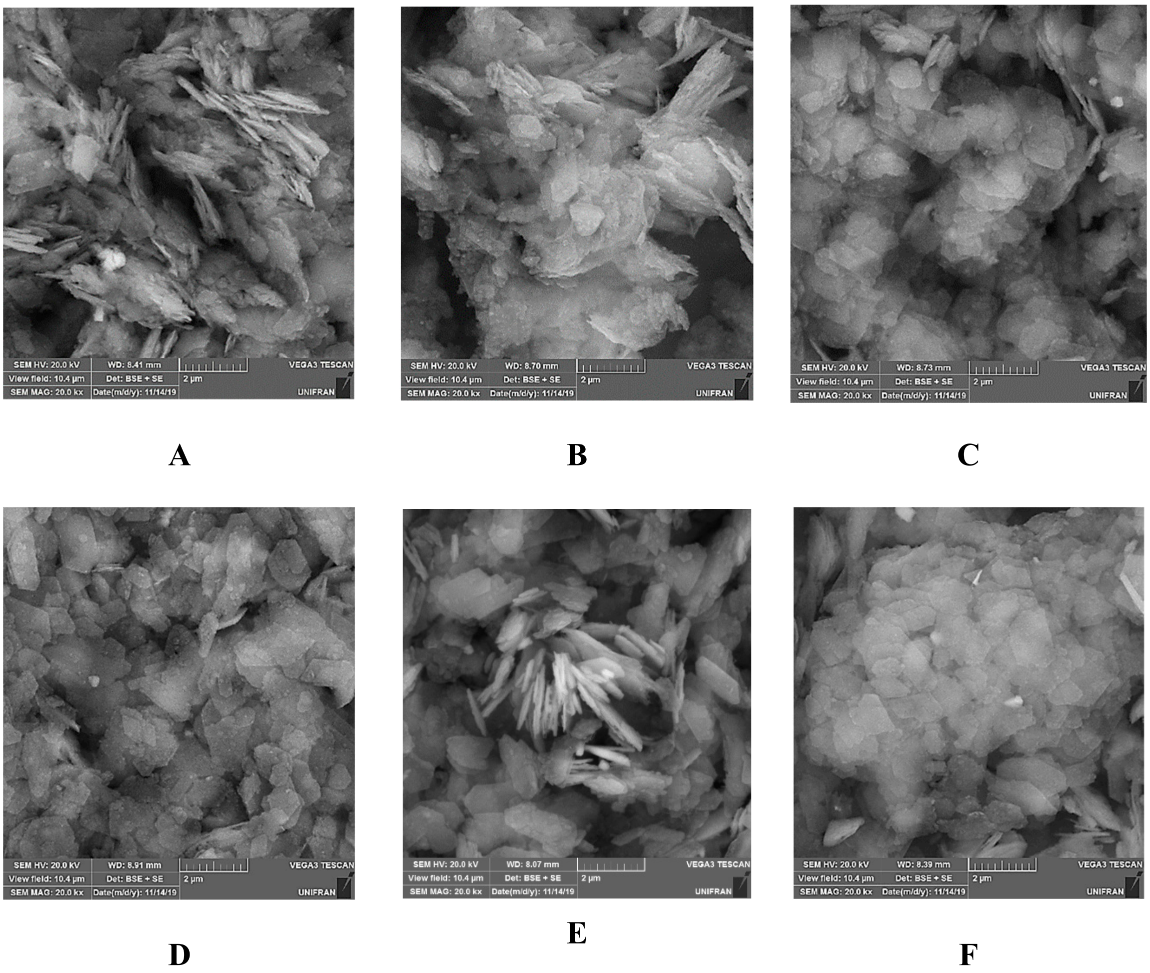

Figure S2. SEM analyses (BSE detector) of Kaol-R (**A**), Kaol-R-DMSO (**B**), Kaol-R-TiO_2 (**C**), Kaol-R-TiO_2-400 (**D**), Kaol-R-TiO_2-700 (**E**), Kaol-R-TiO_2-1000 (**F**). Magnification 30000.

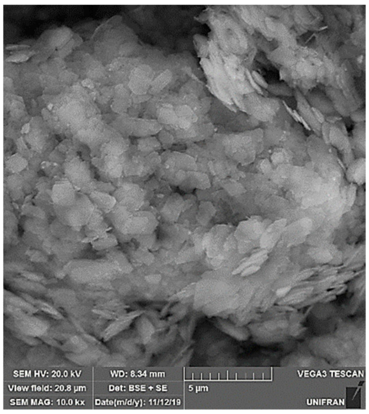

A

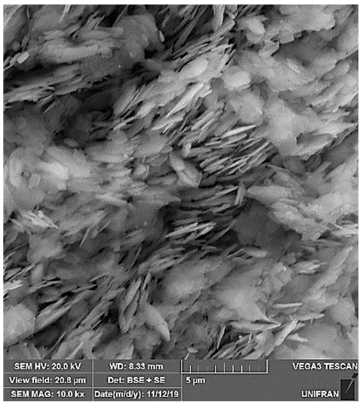

B

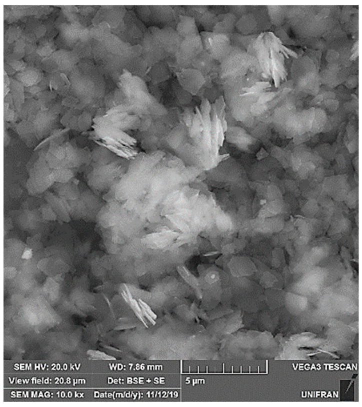

C

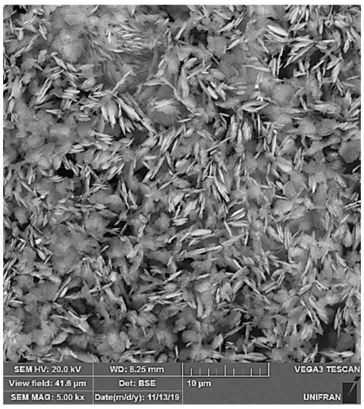

D

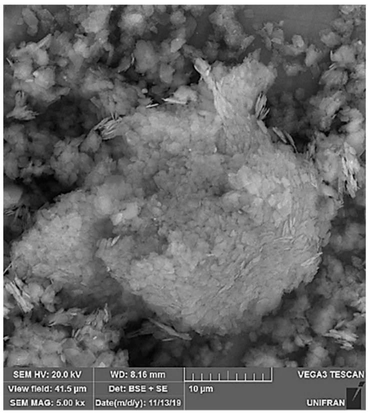

E

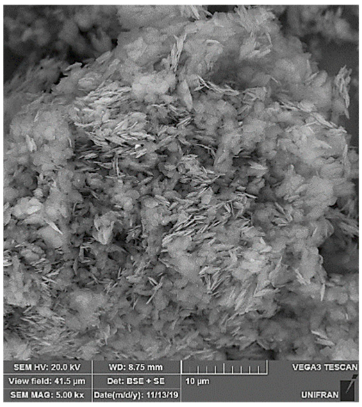

F

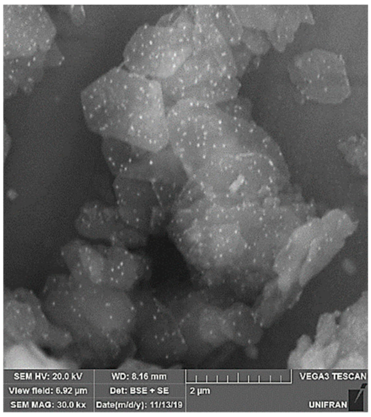

G

Figure S3. SEM analysis (BSE detector) of Kaol (**A**), Kaol-DMSO (**B**), Kaol-TiO_2 (**C**), Kaol-TiO_2-400 (**D**), Kaol-TiO_2-700 (**E**), Kaol-TiO_2-1000 (**F**) (Magnification 5000X) and Kaol-TiO_2-700 (**G**) (Magnification 30000X).

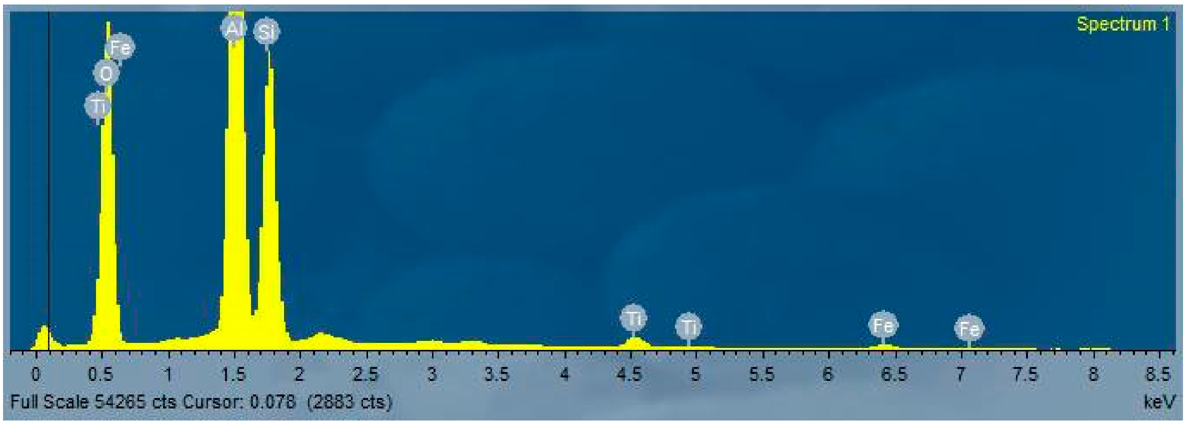

Figure S4. EDX spectrum of Kaol-TiO_2.

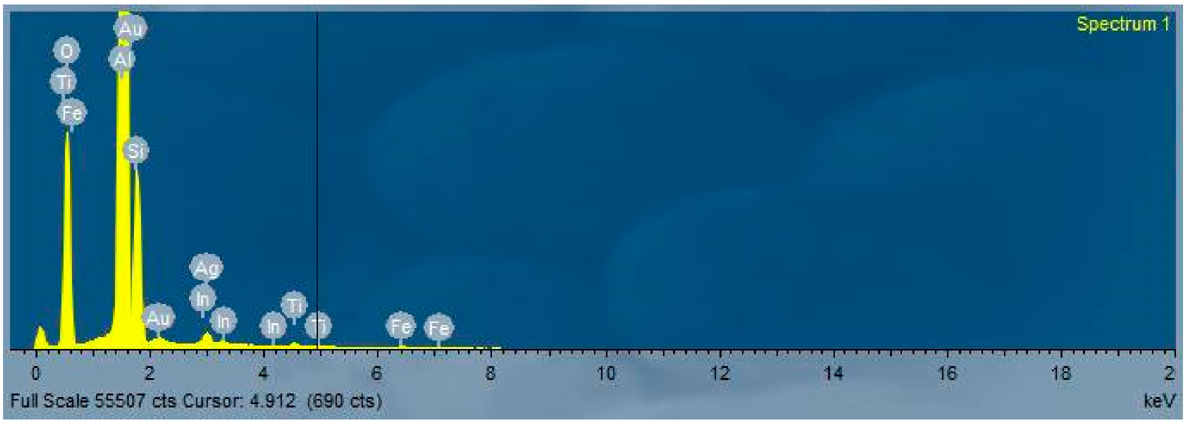

Figure S5. EDX spectrum of Kaol-TiO_2-400.

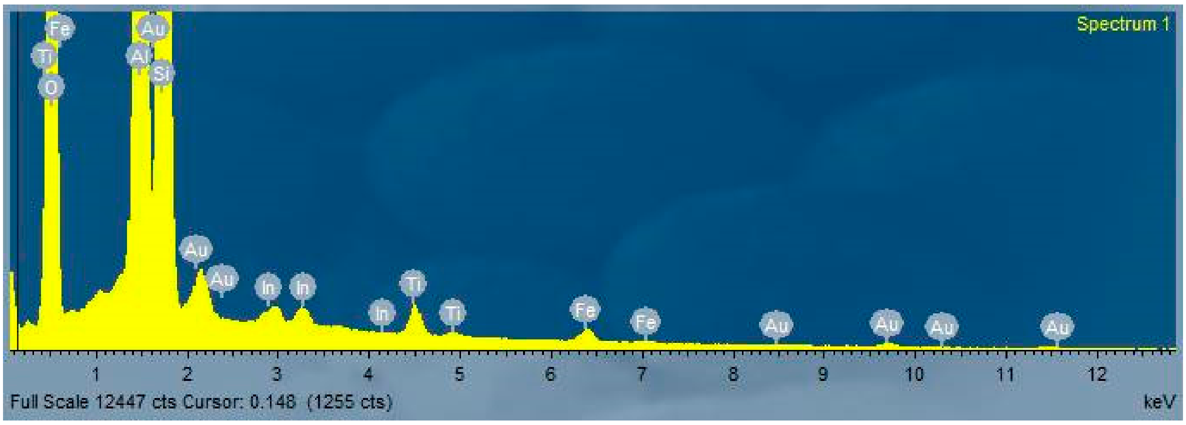

Figure S6. EDX spectrum of Kaol-TiO_2-700.

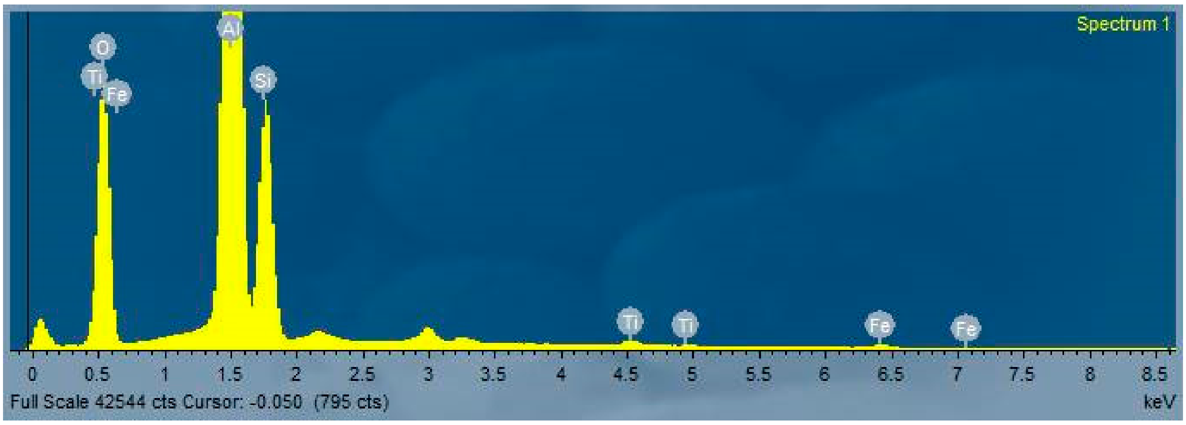

Figure S7. EDX spectrum of Kaol-R-TiO_2.

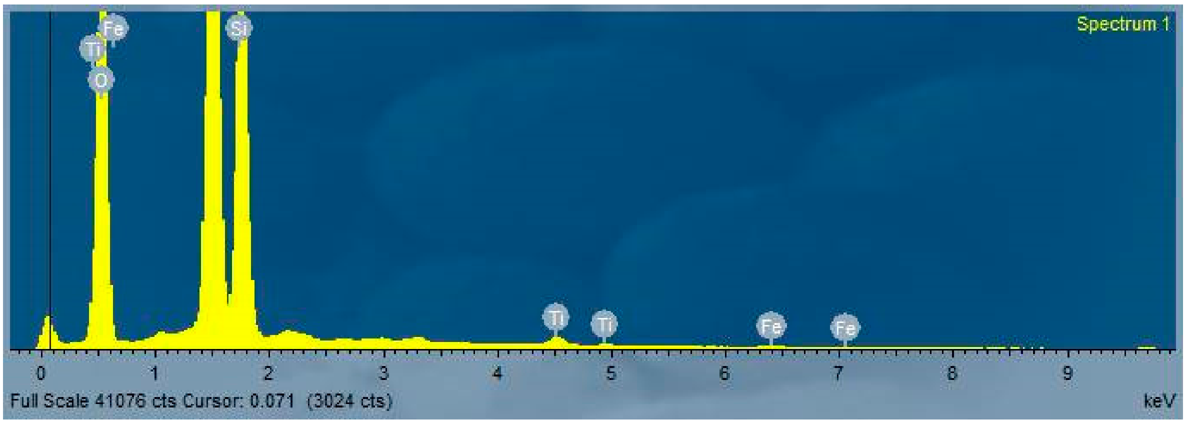

Figure S8. EDX spectrum of Kaol-R-TiO_2-400.

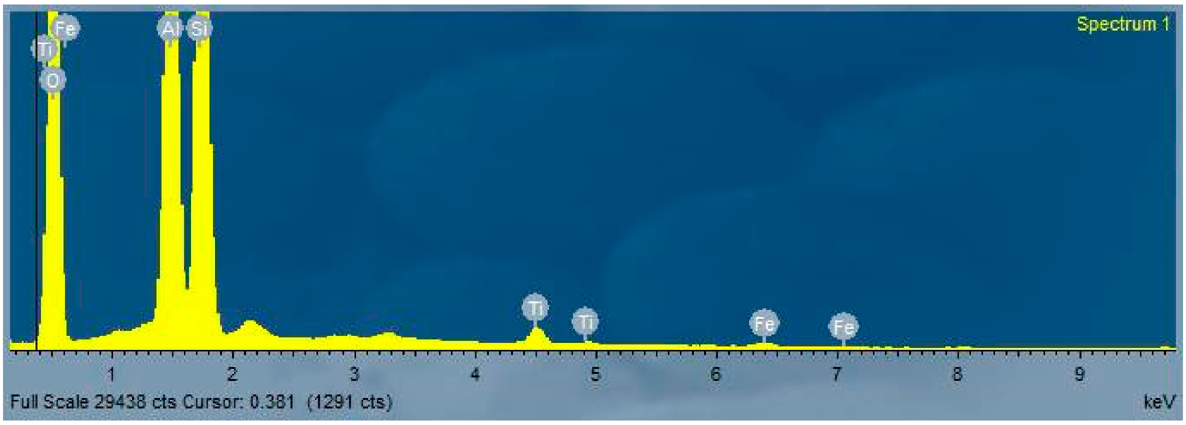

Figure S9. EDX spectrum of Kaol-R-TiO_2-700.

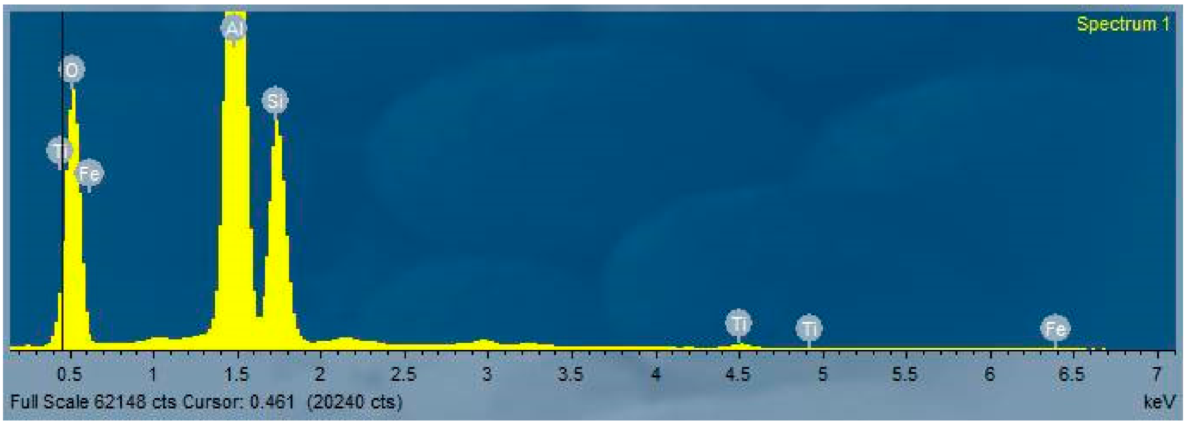

Figure S10. EDX spectrum of Kaol-R-TiO_2-1000.

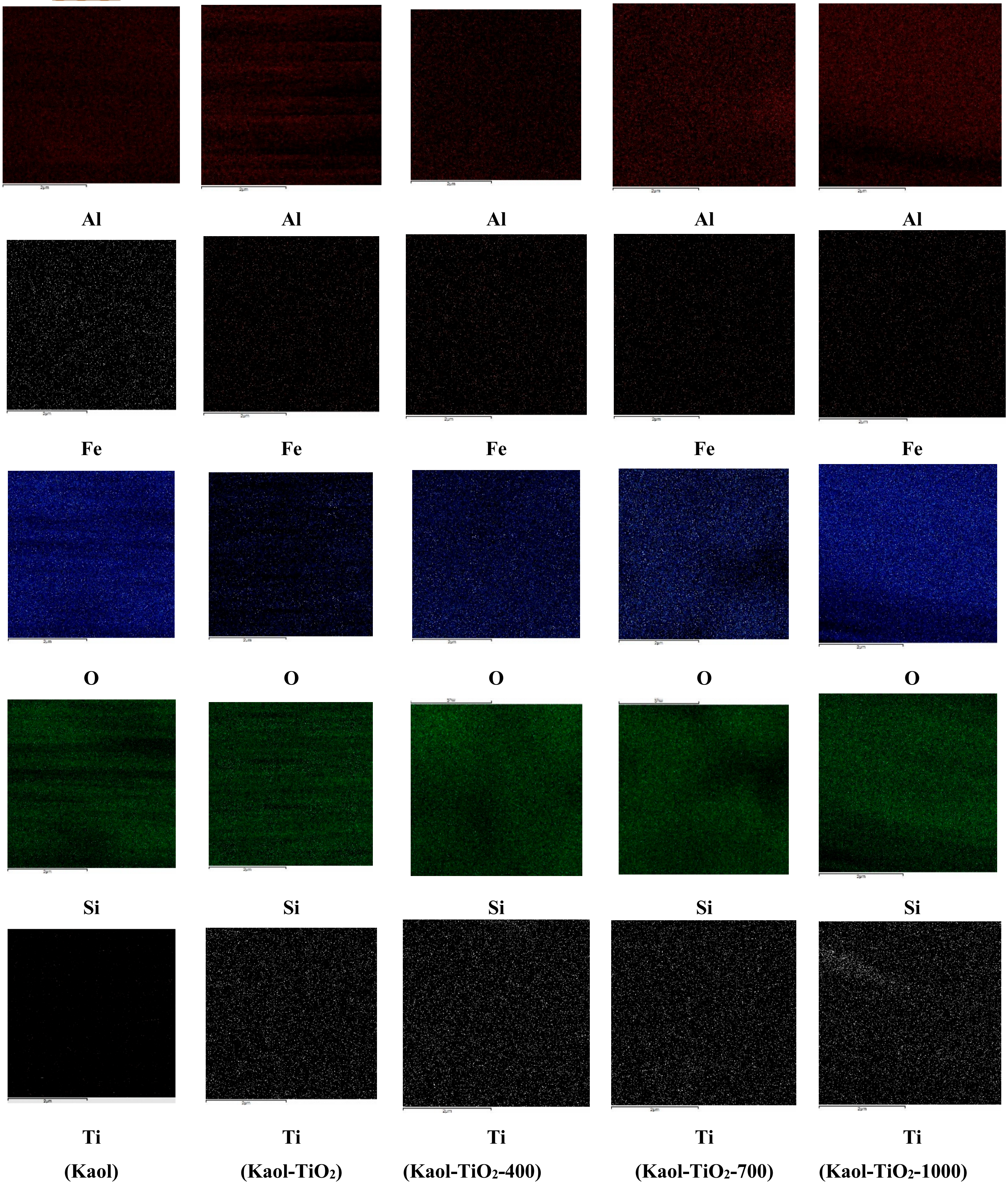

16 **Figure S11.** EDX mapping of Kaol derivatives.

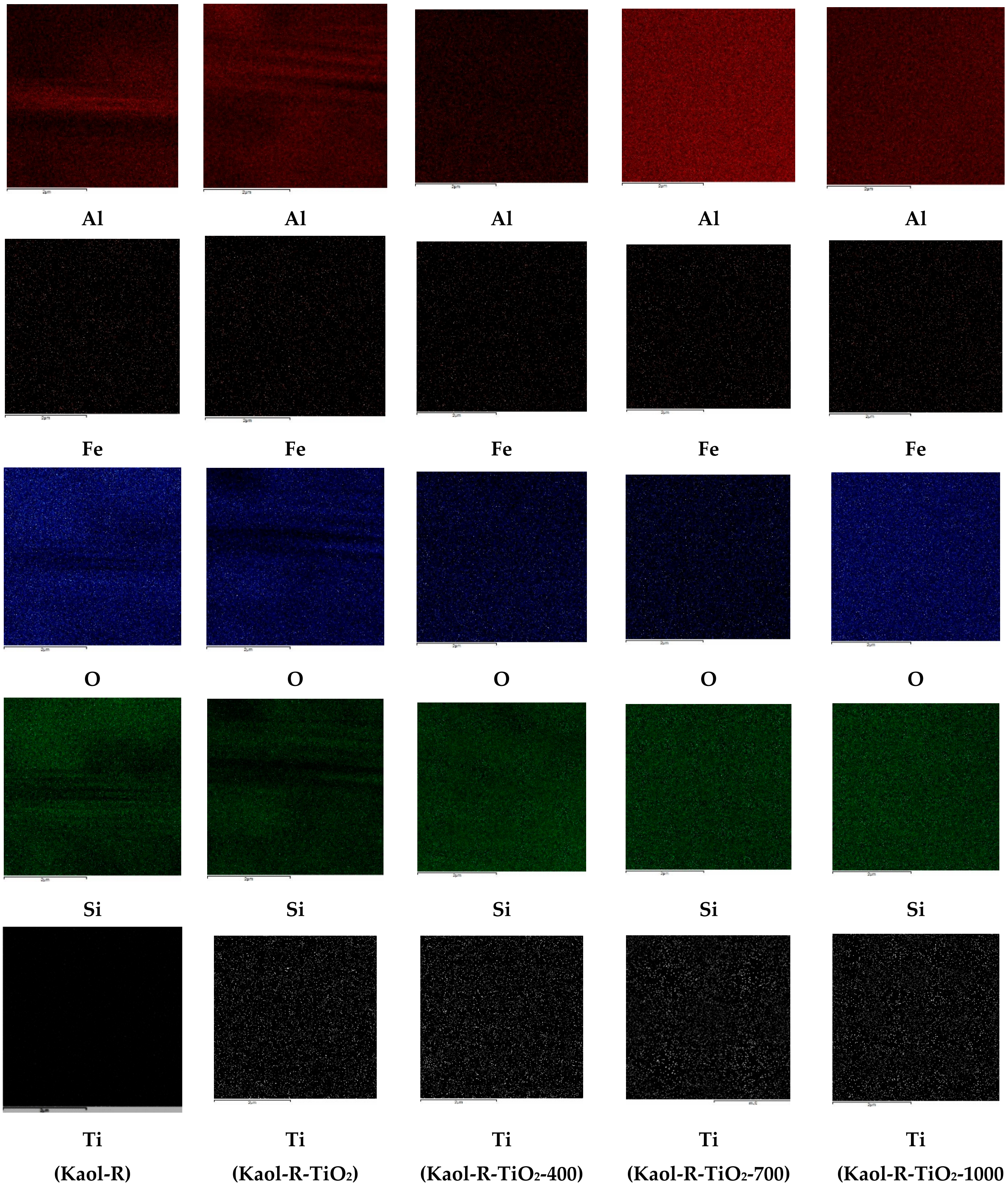

Figure S12. EDX mapping of Kaol-R derivatives.

materials

Table S1. FTIR assignments (cm^{-1}) of the solids derived from white kaolinite.

Assignment	Kaol	Kaol-DMSO	Kaol-TiO_2	Kaol-TiO_2-400	Kaol-TiO_2-700	Kaol-TiO_2-1000
ν $(OH)_{inner}$	3622	3622	3622	3622	-	-
ν $(OH)_{inner\ surface}$	3696, 3666, 3660	3662,3700	3662, 3696	3654, 3696	-	-
δ H-O-H	1628	1652	1650	1654	1620	1622
N Si-O	1026, 1110	1026,1102	1026, 1102	1030, 1108	1072	1088
ν Al-OH_{inner}	916	908	906	916	-	-
ν Al-OH_{inter}	-	962	962	-	-	-
SiO_2 or quartz	792	786	786	792	814	804
δ Si-O-Al	754	744	744	754	-	-
δ Si-O-Si $_{out\ of\ plane}$	698	688	688	698	-	-
Δ Si-O-Al_{oct}	540	554	554	542	-	-
δ Si-O-Si $_{in\ plane}$	470	466	466	472	450	474
N Si-O	430	434	434	430	-	-
S=O- - -HO	-	3504, 3540	3506, 3540	3550	-	-
Ω C–H	-	3022, 2936	3022, 2936	-	-	-

Table S2. FTIR assignments (cm^{-1}) of the solids derived from red kaolinite.

Assignment	Kaol-R	Kaol-R-DMSO	Kaol-R-TiO_2	Kaol-R-TiO_2-400	Kaol-R-TiO_2-700	Kaol-R-TiO_2-1000
ν $(OH)_{inner}$	3622	3622	3622	3622	-	-
ν $(OH)_{inner\ surface}$	3696, 3658	3662, 3700	3662, 3696	3656, 3694	-	-
δ H-O-H	1632	1648	1648	1634	1630	1620
N Si-O	1026, 1108	1034, 1104	1026, 1102	1030, 1110	1088	1074
ν Al-OH_{inner}	916	910	908	916	-	-
ν Al-$OH_{inner\ surface}$	-	960	962	-	-	-
SiO_2 or quartz	792	788	788	792	-	-
δ Si-O-Al	754	746	746	756	-	-
δ Si-O-Si $_{out\ of\ plan}$	696	690	688	698	-	-
Δ Si-O-Al_{oct}	540	554	554	542	-	-
δ Si-O-Si $_{in\ plane}$	470	468	466	472	464	464
N Si-O	430	434	434	430	-	-
S=O- - -HO	-	3504, 3540	3506, 3542	3436	-	-
Ω C–H	-	3022, 2936	3022, 2936	-	-	-

© 2019 by the authors. Submitted for possible open access publication under the terms and conditions of the Creative Commons Attribution (CC BY) license (http://creativecommons.org/licenses/by/4.0/).
